# On the Use of Warm and Hot Water in Surgery

**Published:** 1876-01

**Authors:** Frederick E. Hyde

**Affiliations:** New York


BUFFALO

### Medical and Surgical Journal.

VOL XV. JANUARY, 1876. No. 6.

### Original Communications.

:o:

ART. I.—*Warm and Hot Water in Surgery. A Short Historical Sketch, with the Present Most Approved Methods of Application. With Cases.* By FREDERICK E. HYDE, M. D., New York. [CONTINUED.]

We are indebted to the Germans for the introduction into this country of the *permanent warm-water bath*, or *submersion*. Who, individually, first employed it, I do not know, but at St. Francis' Hospital in this city where I have mostly observed its effects, it has been in use under the direction of Drs. H. F. Guleke and Achilles Rose, during the past five years. The method of its employment having been more systematized since the publication of Dr. Schede's pamphlet in 1871. I am informed also that it has been in use for some time at Mount Sinai Hospital in this city.

We must, however, express our indebtedness to Dr. Frank H. Hamilton for again, after the lapse of twenty-five years, bringing prominently to the notice of the profession in America the benefits to be derived from the use of water in surgery. Dr. Hamilton has given his views and experience upon this subject, accompanied with the details of several cases, in a paper entitled, "On the use of Warm Water, and Especially by Submersion, in Surgery," published in the *Richmond and Louisville Medical Journal*, January, 1874; also in a paper on the "Use of Warm and Hot Water in Surgery, in *The Medical Record*, New York, for May 15, 1874. From these papers I shall quote *in extenso.*

Dr. Hamilton's experience in the use of water, had been with it at a temperature of about 60° F., and with irrigations, following the suggestions of Amussat, but after his connection with St. Francis' Hospital, a German institution in this city, as operating and consulting surgeon, the use of water at a higher temperature was brought to his notice; the beneficial effects of which he had opportunity to observe and appreciate. This experience he put in practice at the Reception Hospitals in this city, of which he was surgeon-in-chief, and also in his wards at Bellevue. From the above institutions Dr. Hamilton has drawn his cases. It will be noticed that the temperature of the water used in the first few cases is about 90° F., but as his experience increased, he raised the temperature to 100° and above, with beneficial results, especially in gangrene. As far as I have been able to ascertain, Dr. Hamilton is the first to use water at the high temperature of 100°, 105°, 110° F. in the treatment of wounds.

In order that these cases may be presented correctly, I will give his own record of them.

Case I.—*Valgus of Great Toe—Excision of Joint.* A laboring man, aged twenty-four, was admitted at St. Francis' Hospital on account of valgus of the great toe of each foot and bunions. The deformity and suffering were such as to prevent him from walking. After consultation with Dr. Rose, it was decided to make a surgical operation. Accordingly on Monday, April 7, 1873, I exposed the bone opposite the metatarso-phalangeal articulation of the great toe on the right foot, and, disarticulation having been effected, I removed three-quarters of an inch of the distal end of the metatarsal bone with the bone-cutter. The wound was quite large, and the incisions laid open the bottom of the foot where inflammatory infiltrations are prone to occur and to do serious mischief.

No dressings were applied, and soon after the operation was completed, the foot was placed in a water-bath at a temperature of about 90° F. The bath was continued, with only occasional interruptions at night, fourteen days, and from this time, and whenever the foot was taken from the bath, the wound was kept wet with linen cloths saturated with water and overlaid with oil-silk. No attempt was ever made to draw the parts together, or to hasten the granulations by other means than the use of water. During the progress of the case it was observed that the foot became gradually œdematous, but the œdema was not accompanied with pain, tenderness, redness or other signs of inflammation. The œdema declined rapidly after removal from the bath, the toe gradually and spontaneously took its natural position, and at the end of four or five weeks the wound was closed, the toe still retaining the power of flexion and extension, and the deformity being entirely removed.

Case II.—*Same as Case* I. On the 2d of June, 1873, Dr. Hamilton operated in the same manner on the opposite foot. The details of the operation and the result are so nearly identical with those recorded in the first operation, that I will not repeat them.

Case III.—*Same as Case* I. A few weeks later Dr. Achilles Rose made the same operation upon an adult male at St. Francis' Hospital. The treatment and results were essentially identical with those obtained in Dr. Hamilton's cases. There was little or no inflammatory reaction; and the toe finally assumed a position of perfect harmony with the other toes.

Case IV.—*Resection at the Elbow-joint.* June 2, 1873, I made resection at the elbow-joint, at St. Francis' Hospital, in the case of a man aged forty, for caries of the olecranon and ulceration of the articular surfaces of the joint. The arm was greatly swollen at the time of the operation, and pus was discharging at various points.

Two long and deep incisions were made at right angles with each other over the olecranon process; the joint was exposed ulcerated and dripping with matter, and the olecranon process was removed at its base with the saw. The bleeding vessels having been secured, but no attempt having been made to close the wound, the arm was at once submerged in a bath of the temperature of about 90° Fah. Almost without interruption the limb remained in the bath during the next fourteen days; until about the tenth day œdema increased steadily along the arm and fore arm, but at this time it began to abate even before the limb was removed from the bath, subsequently it was treated with water applied by means of folds of cotton cloth covered with oiled silk. The wound is now almost closed, and the limb is reduced to nearly its natural size. Flexion and extension are quite free through a space of eight or ten degrees, and motion is unaccompanied with pain.

From the day of the operation to the present moment there has been no sign of inflammatory reaction either in the limb itself or in the condition of the system. The patient has eaten and slept well all the time.

Case V.—*Lacerated Wound.* On the 30th of June, 1873, James Austin, aged eleven, was admitted to the Ninety-ninth Street Reception Hospital, his leg having been caught between a street car and a loaded wagon while both were approaching each other rapidly. The soft parts above the heel were torn away extensively, making a wound nearly large enough to receive the hand; the tendo-Achillis was gone, the muscles behind the malleolus internus were severed or crushed, the posterior tibial artery was divided, the malleolus externus broken, and the tibio-tarsal articulation completely exposed.

I decided not to amputate, but the boy being exceedingly restless and impatient of treatment, it was thought impracticable to use the bath, Having therefore tied the posterior tibial artery, the limb was laid upon a pillow covered with oil-cloth, and completely enveloped with two thicknesses of sheet lint previously saturated with tepid water, and then enclosed with oil-silk. Great care was taken to cover the toes, foot and leg completely with these water dressings. Essentially this plan of treatment was continued until the wound was closed. On the thirtieth day after the injury a special note of the case was made. The foot was œdematous below the wound, but there was no œdema or swelling from any other cause in the leg above the wound. The patient was quiet, the wound suppurating and granulating finely, but the joint surfaces remained completely open, and could be seen free from vascularity or other signs of inflammation.

September 29th he left the hospital with the wounds entirely closed.

Case VI.—*Lacerated and Contused Wound of Foot.* July 29, 1873, Patrick Evers, aged thirty, was received at the Park Reception Hospital, having a few hours before been injured by a locomotive on the New Jersey Central Railroad. The right foot was crushed, and the bones more or less broken nearly in a line with the metatarso-phalangeal articulation. To be more precise, for this is a most extraordinary case in regard to results, the little toe was cut nearly off and broken, the fourth metatarsal bone was comminuted, the first phalanx of the great toe was broken, the integument over the joints was discolored, somewhat torn and swollen, and the bottom of the foot, at a corresponding point, was extensively lacerated The man had been drinking heavily, and was not yet sober, but he complained greatly when the foot was handled.

Being called in consultation, I said to Dr. Fluhr, the hospital surgeon, whose opinions always commanded my respect, "What do you think; can we save the foot?" To which he replied, "In every case similar to this in which an attempt has been made to save the limb, the patient has died." I added "and my experience has been the same, but I have never seen the trial made by submersion, and I have great faith, from my late experience, that it will succeed." It was decided to try this method. I therefore ordered a bath from Otto & Reynders, and the limb was submerged in water at a temperature of about 100° Fah. He soon complained of this as being too warm, and as causing a burning pain which extended up to the knee. By the addition of ice the temperature was lowered to about 90° Fah. The thermometer was not used, and I cannot therefore be more precise in my statement.

On the 31st July I found the foot considerably swollen, but not painful. He was resting quietly. Five days later I discovered that the gangrene was extending along the top of the foot and beyond the parts originally injured. The circumstance was unfavorable, and I began to fear that we had made a mistake in attempting to save the foot; but placing my hand in the water, I found that it felt cool, and it was at once suggested that the low temperature of the water was the probable cause of the mischief. The orderly admitted that he had neglected to attend to this latterly with as much diligence as at first. Hot water was introduced and the temperature again elevated to about 90°. Two days later the gangrene was arrested, and the line of demarcation had begun to be formed. On the sixteenth day the foot was removed from the bath; and from this time, until he left the hospital, it was treated with water fomentations alone. Shortly after its removal from the bath a small abscess formed in the sole of the foot, which, being opened, soon healed. It is quite probable that if it had been kept submerged longer this would not have happened.

September 10th, less than six weeks after the accident, he was sent to Bellevue as a convalescent, with the foot nearly healed, and with the loss of only three toes.

Case VII.—*Necrosis of the Humerus.* July 21, 1873, ———, aged forty, was received at St. Francis' Hospital; I made an incision five inches in length upon the front of the arm a little below its middle, exposing the bone, and with the aid of the trephine and gouge, removed several large central sequestra. The position of the wound rendered it difficult, although not impossible, to use the bath. It was therefore covered with folds of cotton cloth saturated with water, only a pledget of loose lint being laid in the chasm caused by the removal of the bone. The whole was surrounded with oiled silk, and the limb laid upon a pillow.

July 31st. No reaction nor swelling had occurred, and the wound was granulating finely.

September 5th. Wound not yet closed, but slowly closing. Water dressings are still continued.

Case VIII.—*Lacerated Wound and Fracture of Leg.* Jacob Habers, aged twenty-five, was run over by a street car, August 9, 1873, breaking the fibula of the right leg and causing an extensive laceration of the soft tissues. During the third or fourth day succeeding the accident, the lacerated wounds were dressed with oakum bound upon the limb with a roller. August

13th he was received at St. Francis' Hospital, the whole thigh and leg being greatly swollen, and his general condition exceedingly feeble. At a consultation it was decided that neither the state of his limb nor of his general system would warrant amputation, but that his chance of recovery without amputation was small. Water fomentations were at once applied, as he was too feeble and the leg too much swollen to admit of the use of the bath. These were continued two weeks, when the bath was substituted; the bath being employed, with only occasional interruptions, during the next five weeks. He then returned to the fomentations.

October 1st. The wound is nearly healed, and the patient is completely restored to health.

November 1st. Wound closed.

Case IX.—*Lacerated and Contused Wound of Foot, with Fracture.* Richard Shay, aged forty, admitted to Ninety-ninth Street Reception Hospital, September 17, 1873, having just received a severe injury of the left foot caused by the fall of a heavy stone upon it. The first and second toes, including the lower portions of the corresponding metatarsal bones, were crushed and broken. The other toes, with considerable portions of the top and sides of the foot, were badly bruised. I did not see him until the third day, at which time the condition was such as ordinarily had been thought by me to demand amputation, since the attempt to save the limb under the same circumstances has generally resulted in death.

The limb was at once submerged in water at the temperature of 90° Fah. No other dressings employed.

September 30th. Found patient with foot still in the bath. Great toe and adjoining toe sloughing off. No extension of gangrene beyond this. Limb very œdematous as high as the tubercle of the tibia. Very little pain or tenderness. No febrile disturbance. Sleeps well. I directed a teaspoonful of common salt to be added to the water every two or four hours, or as often as the hot water is added. I did this at the suggestion of Dr. Rose, hoping to contract the tissues and diminsh the œdema.

October 2d. Œdema not diminished. Patient feels well, and everything appearing favorable, so far as the foot is concerned, it is removed from the water and dressed with water fomentations.

November 1st. Wound closed and patient discharged.

Case X.—*Comminuted Fracture of Leg followed by Traumatic Gangrene.* Amputation. (Full notes of this case were not obtained, but as it presents a new point of interest in relation to the water treatment, Dr. Hamilton gives a brief report of it.)

A German, about thirty years of age, of temperate habits, was run over by a street car, October 24th, the wheel traversing the left leg a little above the ankle. When admitted to the Ninety-ninth Street Reception Hospital soon after the accident, it was ascertained by Dr. Delgado, our experienced House Surgeon, that the tibia and fibula were broken and comminuted, and believing that it required amputation, a message was sent to me. Through some inadvertance the message did not reach me until night, probably eighteen hours after the injury was received. When I arrived the gas had given out, and the amputation was deferred until morning. My second visit was made nearly thirty-six hours after the receipt of the injury, and it was then apparent the favorable period for amputation had passed. The leg was swollen, red and tender as high up as the knee. The intermediate period of inflammation had fairly set in, and it was believed that we should give him the best chance for life by delay, or by tiding him over into the "secondary" period or period of suppuration. There were several reasons which controlled this decision. First, the fact that operations in the period of inflammation are generally fatal. Second, sensation in the foot and toes was only a little less than natural; the integument was nowhere broken, and there seemed to be some small ground of hope that the foot might be saved. Third, I had great confidence in the power of warm water to control inflammation and limit gangrene.

The limb was too much broken to admit of submersion, it was therefore laid upon a pillow covered with oilcloth, and cloths saturated with *hot* water were applied to the entire foot and leg. These were enclosed in cotton batting, and the cloths and hot water were directed to be renewed every hour. The directions were carried out fully, but the vitality of the foot gradually diminished, and gangrene set in fairly by the second or third day. No change was made in the treatment, and on the ninth day after the injury the gangrene had implicated all the parts originally injured by the wheel of the car, and the foot was insensible, but there was no extension of gangrene above the line traversed by the wheel; the inflammation and swelling had entirely disappeared, and a complete line of demarcation was formed, by suppuration, between the living and dead tissue. The patient's pulse was slow and soft, but he suffered considerable pain in the limb, and was desirous that it should be amputated. The period seemed favorable, and I amputated about five inches above the knee. Incidentally I will say that the amputation was made according to the plan recently suggested and practiced by Esmarch, binding the limb firmly from the toe up with a roller and without a tourniquet. The amount of blood lost was less than I have ever seen in an amputation at the same point.

CASE XI.—*Traumatic Gangrene Arrested by Hot Water Submersion; Spontaneous Separation of Foot and Leg on Ninth Day after Submersion; Recovery.* Hugh Quigley, aged twenty-six, admitted to Ninety-ninth Street Reception Hospital, December 8, 1873, with a fracture of the right leg, caused by the fall of a heavy stone upon the leg about one hour before admission. The bones were broken near the junction of the lower and middle thirds. There was a slight laceration on the inside of the leg, the posterior tibial artery pulsated distinctly. Dr. Delgado, the House Surgeon, applied a roller and placed the limb in a box padded with oakum.

December 9th. A padded leather splint was applied to the outside of the leg and secured by a roller.

December 10th, 4:30 P. M. Foot found to be cold, congested and œdematous; pulse 130; splint and bandage removed, and limb wrapped in cotton batting, with hot bottles.

December 11th. I was summoned. There was then no sensation below the point of fracture; the pulsation of the posterior tibial had ceased; the leg was swollen and erysipelatous to a point some inches above the knee, and gangrene had extended in patches to about the middle of the leg. It was apparent that the condition of the limb and of the patient were in the highest degree unfavorable for an amputation, which, in case it were made, must be above the knee. Surgeons need not be reminded how seldom an amputation at this period (intermediate), and under these circumstances is successful.

Dr. Delgado, at my suggestion, made several incisions through the gangrenous tissues, and at once placed the limb in a water bath at a temperature of 110° Fah. No medicine.

December 12th. Pulse 112; temperature 103½° F.

December 13th. Pulse 119; temperature 99¾° F.

December 14th. Pulse 114; temperature 101¼ °F.

Swelling of limb above the fracture increasing. Permanganate of potassa and chloride of zinc put in the water as disinfectants. Stomach rejects food; bowels are constipated; appetite poor. Biscarbonate of bismuth ʒi. ter die.

December 15th. Pulse 104; temperature 97¾° F. Vomiting has ceased. I saw him to-day for the first time since the limb was submerged. The water in the bath has been changed twice daily. The patient seems cheerful; says he sleeps very well, has no pain in the limb, nor is it particularly tender upon pressure above the line of gangrene. I cannot see that the gangrene has extended at all since the present treatment was adopted.

December 16th. Pulse 90; temperature 98° F.

December 17th. Pulse 96; temperature 99° F.

December 19th. Erysipelas has disappeared, and the line of demarcation is formed near the junction of the lower and middle portions of the leg. This is the eleventh day since the injury was received, and the eighth since submersion was employed. With my forceps and scissors I proceeded to trace the separation, and soon found that all the dead tissues were separated, except a portion of the gastrocnemius and soleus, which latter I divided with a knife low down, and the limb fell off at the point of fracture; the incisions of these muscles causing only a moderate hæmorrhage from very small vessels. These I tied; neither anæsthetics nor the tourniquet were employed. The stump was then covered with warm water dressings, supported by a snug roller.

December 21st. Pulse 92; temperature 100° F. Leg again immersed in warm water.

December 25th. Limb removed from bath. Granulations abundant.

December 28th. Balsam of Peru substituted for water as a dressing.

December 30th. A small abscess was opened which had formed near the knee; discharged about three ounces of pus.

January 24, 1874. I removed the projecting and exfoliated portion of the tibia with my fingers.

January 31st. Patient sits up in his chair two hours daily, and the stump is closing rapidly. It is quite probable, however, that at some future time it will be necessary to resect portions of both the tibia and fibula, in order to give a proper shape to the stump. I have since made resection and the stump is nearly well.

Case XII.—*Lacerated and Contused Wound of Foot, with Fracture of Bones, Caused by a Car Wheel; Gangrene and Recovery.* Bernard Monahan, aged forty-five, admitted to Ninety-ninth Street Reception Hospital, January 11, 1874, with a compound fracture of the left foot, caused by its having been traversed by a street car a short time previous to admission. The foot below the tarsus was much larcerated, and several of the bones were broken. The right foot was also slightly injured.

On the following day I was summoned to decide the question of amputation. It was determined to employ submersion, in the case of the left foot, with water at a temperature of 100° Fahrenheit. The right foot was enclosed in water-dressings and placed in bed.

January 19th. Left foot œdematous, but not tender or painful. The second and third phalanges of the second toe have dropped off.

January 20th. Patient ordered extras, as food and tonics. Foot taken from bath at night.

January 31st. Right foot is well; left foot is granulating and cicatrizing; water-dressings discontinued and Balsam of Peru substituted.

The condition of the pulse has been recorded each day, and at no time during the progress of the case has it exceeded 80 in the minute. There has been no fever.

Case XIII.—*Excision of the Head of Metatarsal Bone of Great Toe for Valgus Hallux.* Adult male—St. Francis' Hospital.

*Operation.*—February 2d, 1874. The patient having been placed under the influence of ether, Dr.Scharlau applied Esmarch's bandage from the toes to the middle of the leg. I then made a crucial incision over the lower portion of the metatarsal bone, rather upon its inner and dorsal aspect. In attempting to pass the chain-saw it was broken, and the section of the bone was made with the bone cutter, in consequence of which the bone was divided irregularly. After disarticulation of the head the bursa was found to communicate with the joint, and the joint surfaces were slightly carious. During the operation not one drop of blood appeared in the wound, but there was a slight oozing of *serum,* which required to be removed once or twice by the sponge. The ligature remained upon the leg about twenty minutes, and when it was removed, quite a free bleeding occurred, which, however, was promptly arrested by the application of carbolic acid water—ten drops of the saturated solution to an ounce of water.

In order to avoid secondary hæmorrhage, the foot was treated with cool-water dressings until the following morning, when submersion in warm water was substituted, and this was continued about two weeks. One small abscess formed and opened upon the top of the foot, about two inches from the wound. In all other respects the progress of the recovery was uninterrupted. March 20th, the wound was healed.

Case XIV.—*Valgus Hallux—Excision of Metatarsal Bone of Great Toe.* Male, aged eighteen, admitted to St. Francis' Hospital February, 1874, with valgus hallux in both feet, which had existed several years. The right foot troubled him most, the integument over the articulation being almost constantly inflamed. It was decided to practice excision only upon this foot.

*Operation.*—February 9th, 1874, assisted by Drs. Rose and Scharlau, the patient was anæsthetized, and Esmarch's bandage applied. The incisions were the same as in the preceding cases; no blood appeared in the wound until the Esmarch bandage was removed; the blood then flowed quite freely, but in a few minutes it ceased *spontaneously*, the total amount of blood lost not exceeding two or three ounces.

The wound was treated with cool-water dressings until the following morning, when the warm-water bath was substituted. The bath was used about fourteen days, and warm-water fomentations were then substituted. March 20th, the wound was closed, and the patient was walking about, the progress of the case toward recovery having been from the first uninterrupted.

Case XV.—*Lacerated Wound of Hand—Submersion on the Seventh Day—Death on the Thirteenth.* Andrew Bedhman, aged twenty-seven, admitted to Bellevue Hospital, Dr. Wood's service, January 12th, 1874. His left hand had been caught in a "moulding machine" a few hours before admission, and was "terribly lacerated—in short, all torn to pieces." By advice of Dr. Wood, the acting house surgeon proceeded at once to amputate the first and third fingers through the middle of the metacarpal bones, and the second finger at the second joint. Only one or two vessels required the ligature. The hand was then laid upon a splint and dressed with carbolic-acid solution and lint.

January 16th. A consultation was called by Dr. Wood, at which I was present. Hand greatly swollen and wounds gaping, foul and gangrenous: redness and swelling extending above the wrist pulse in the morning of this day, 100; in the afternoon, 120; temperature, 106° F.; respiration, 20; patient very despondent. The period and condition seemed peculiarly unfavorable for amputation, and it was decided to employ warm-water submersion. We subsequently learned that no bath could be obtained until the 19th. On this day (16th) he took quin. sulph., grs. x., at 6 and 9 P. M., also tr. aconite, Fleming, minums ij, at 6, 7, 8 and 9 P. M.

On the 18th he had a slight chill.

January 19th. "Hand begins to look a little better; some healthy discharge" but most of the wound is "still foul and offensive." House surgeon ordered sodæ sulphitis, grs. xv., and quin. sulph., grs. v., ter in die.

January 20th. Some improvement in the appearance of the granulations; pain in shoulder.

January 22d. Opening made above the wrist to let out pus. 23d. Explored again for pus, but none found. 24th. For the first time, the submersion has been continued all night.

January 25th. At 5 A. M. the night watchman, in making his rounds, discovered that the hand was bleeding, "which was arrested by the orderly before the acting house surgeon could arrive (which was in two minutes). Patient had lost very little blood (water in bath hardly tinged), but was terribly frightened." He was informed that he had lost very little blood, but from this moment he failed rapidly, and died about three hours later.

REMARKS.—The records of the autopsy cannot be found, but it is understood that there was no evidence of pyæmic infection. The period at which submersion was commenced—the sixth day—renders it apparent that the water treatment had little or no responsibility for the final result. It is my opinion, founded upon my experience in similar cases, that submersion, practiced from the first or second day after the accident, would have saved his life.

The following is the record of temperature and heart's action from and after the fourth day:

January 16th. (Day of consultation). P. M. Temperature, 106°; pulse 120.

January 17th. (Fomentations commenced). P. M. Temperature 106½°; pulse 108.

January 18th. (Slight chill).

January 19th. (Submersion commenced to-day). P. M. Temperature 106°; pulse 120.

January 20th. Temperature, 103½°; pulse 112.

January 21st. Temperature 106°; pulse 112.

January 22d. (Incised for pus). Temperature 103°; pulse 112.

January 23d. (Explored for pus; none found). Temperature 100¼°; pulse 112.

January 24th. (In bath all night).

January 25th. Died at 8.20 A. M.

TO BE CONTINUED.

